# Soybean Yield Formation Physiology – A Foundation for Precision Breeding Based Improvement

**DOI:** 10.3389/fpls.2021.719706

**Published:** 2021-11-15

**Authors:** Jonathan T. Vogel, Weidong Liu, Paula Olhoft, Steven J. Crafts-Brandner, Joyce C. Pennycooke, Nicole Christiansen

**Affiliations:** BASF Corporation, Research Triangle Park, NC, United States

**Keywords:** soybean, yield, genome editing, precision breeding, crop growth rate, leaf area duration, seed filling period, duration of flowering

## Abstract

The continued improvement of crop yield is a fundamental driver in agriculture and is the goal of both plant breeders and researchers. Plant breeders have been remarkably successful in improving crop yield, as demonstrated by the continued release of varieties with improved yield potential. This has largely been accomplished through performance-based selection, without specific knowledge of the molecular mechanisms underpinning these improvements. Insight into molecular mechanisms has been provided by plant molecular, genetic, and biochemical research through elucidation of the function of genes and pathways that underlie many of the physiological processes that contribute to yield potential. Despite this knowledge, the impact of most genes and pathways on yield components have not been tested in key crops or in a field environment for yield assessment. This gap is difficult to bridge, but field-based physiological knowledge offers a starting point for leveraging molecular targets to successfully apply precision breeding technologies such as genome editing. A better understanding of both the molecular mechanisms underlying crop yield physiology and yield limiting processes under field conditions is essential for elucidating which combinations of favorable alleles are required for yield improvement. Consequently, one goal in plant biology should be to more fully integrate crop physiology, breeding, genetics, and molecular knowledge to identify impactful precision breeding targets for relevant yield traits. The foundation for this is an understanding of yield formation physiology. Here, using soybean as an example, we provide a top-down review of yield physiology, starting with the fact that yield is derived from a population of plants growing together in a community. We review yield and yield-related components to provide a basic overview of yield physiology, synthesizing these concepts to highlight how such knowledge can be leveraged for soybean improvement. Using genome editing as an example, we discuss why multiple disciplines must be brought together to fully realize the promise of precision breeding-based crop improvement.

## Introduction

Improvements in crop yield led to the establishment of modern human societies. These improvements continue to this day, as there is pressure to produce greater yield with fewer acres using even more sustainable agronomic practices. Advances from all corners of the plant biology community are required to realize continued gains. Toward this goal, an understanding of crop yield physiology is essential to fully apply genetic, molecular, or biochemical knowledge to improve the physiological processes underlying crop yield formation. This is of particular importance for precision breeding approaches such as genome editing, where target genes impacting physiological processes are required. As soybean is a major crop of societal importance, it represents a good model for discussing yield formation and improvement.

Soybean (*Glycine max* (L.) Merr.) is a key global commodity and the leading oilseed crop produced in the world. It is an integral part of multiple food, feed, and industrial products. As compared to corn seeds, which are largely starch, soybean serves as an important protein and oil component for animal feed and human consumption. Soybean represents more than 60% of global vegetable oil and protein consumption and is the fourth largest field crop by volume (U.S. Department of Agriculture, Agricultural Research Service (USDA), 2019). Continual increases in soybean genetic potential are critical to meet global demands for plant-based protein and oil, as per capita soybean consumption is projected to increase ∼17% by 2029 (OECD and Food and Agriculture Organization of the United Nations, 2020). As such, a continuous increase in soybean yield is important not only for growers and animal producers, but also for consumers and global agricultural sustainability.

**FIGURE 1:**
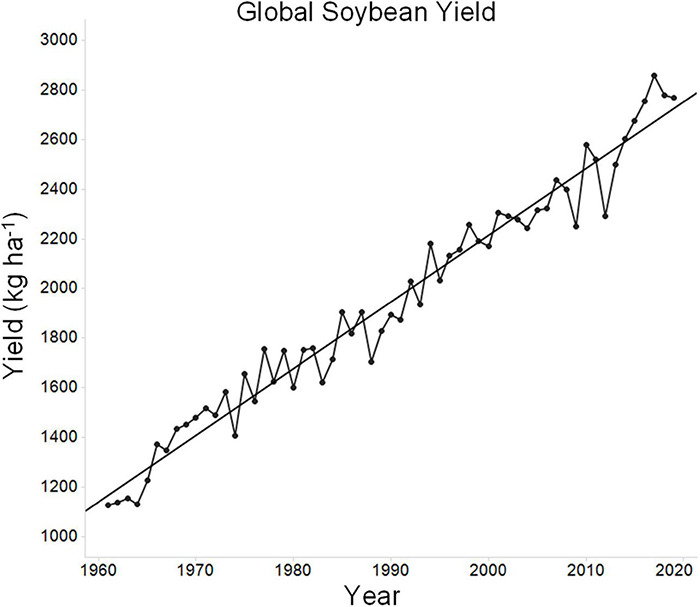
Average global soybean yield over time. Data for average historic global soybean yield was sourced from the UN Food and Agriculture Organization (FAO) database in September 2021 (http://www.fao.org/faostat/en/#data). The figure depicts average soybean yield over time and a regression line was plotted for reference.

Over the past 60 years, global soybean yield has increased progressively (Figure 1). During this time, the global average soybean yield has increased from ∼1,128 to ∼2,769 kg ha^–1^. Much of this improvement is due to improved genetics, as modern cultivars clearly yield more than older cultivars (Rincker et al., 2014). The theoretical maximum yield of soybean has been reported to be in the range of ∼7,250–11,000 kg ha^–1^ (Specht et al., 1999; Sinclair et al., 2004; Van Roekel et al., 2015). This maximum, which is the yield that can be obtained in the absence of stress, is the yield potential. While this estimate can be debated, as there are indeed records of much higher yields in small plot yield contests (12,777 kg ha^–1^), university researchers using optimal growing conditions including irrigation rarely exceed 6,725 kg ha^–1^ (Van Roekel and Purcell, 2014; Winsor, 2021). Yields of 6,725 kg ha^–1^greatly exceed average yields (for example, 2019 average soybean yields in the top three soybean producing countries were 3,189 kg ha^–1^ for the United States, 3,185 kg ha^–1^ for Brazil, and 3,334 kg ha^–1^ for Argentina) and thus it can be argued that soybean intrinsic yield potential is already high and that growth limiting factors, largely stress, are the major limitations to high yield across the broader regions of soybean production. Such limits on growth stem from both biotic and abiotic factors, including suboptimal agronomic practices. Of the potential stresses, water and temperature are two of the most important factors (Dornbos et al., 1989; Gibson and Mullen, 1996; Specht et al., 1999; Ergo et al., 2018; Gajić et al., 2018; Jumrani and Bhatia, 2018). Soybean yield can be significantly decreased upon exposure to temperature stress, water stress, or the combination of both stresses together (Veas et al., 2021). Tolerating or resisting these stresses is critical for crop improvement. For example, over a 25-year period in Nebraska, soybean yield improvement has occurred at a significantly higher rate under irrigated conditions as compared to non-irrigated conditions (Specht et al., 1999; Mekonnen et al., 2020). This highlights the impact of environmental stress on the progress of plant breeding. Any lines developed specifically to improve stress tolerance require yield performance both during stress and in the absence of stress, as growers will not tolerate lower yield under favorable growing conditions (Sinclair and Purcell, 2005).

Crop yield is reliant on the sum of photosynthetically active radiation (PAR) absorbed by the crop over the course of a growing season and subsequently converted into harvestable grain yield (Rattalino Edreira et al., 2020). Efforts to increase yield must improve underlying physiological processes that allow the crop community to best utilize this energy in expressing its yield potential by creating and filling seeds while also mitigating environmental stress. Yield is a multigenic trait, impacted by the contribution of many loci across the genome for various physiological, abiotic, and biotic stress tolerance factors that all interact over time during the growing season to determine final yield. This genetic complexity is further increased via genotype (G) interactions with both the environment (E) and agronomic management (M) practices (G × E × M). Due to the quantitative nature of yield, it is helpful to better understand the physiological parameters with known relationships to yield.

Here, we consider yield from a top-down perspective as we discuss the main physiological factors important for yield. Specifically, we focus on the physiological parameters with definitive evidence for a relationship to soybean yield in the field. This field-based focus is important, as yield is defined on a per unit area basis. Physiological knowledge is critical, as crop physiology serves as a basis for understanding how soybean yield has improved and informs future yield improvement efforts.

## An Overview of Soybean Development

**FIGURE 2:**
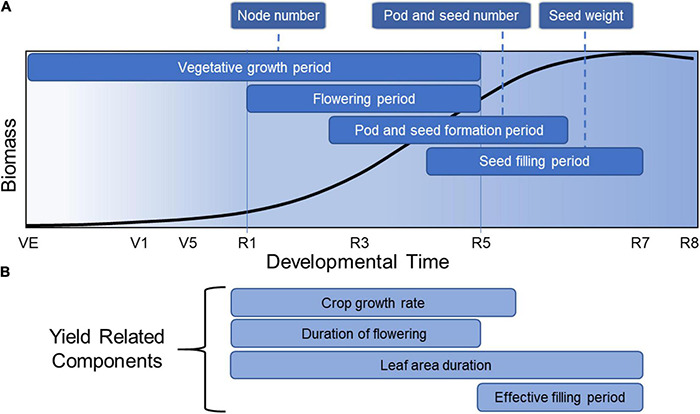
Total soybean biomass accumulation over developmental time overlain with the critical periods for yield formation. This figure represents the general accumulation of soybean biomass accumulated in all parts of the plant over developmental time, adapted from data described by Pederson and Licht (2014). **(A)** Critical periods for node, pod, seed number and seed weight are indicated. **(B)** Yield-related components are indicated during the developmental times at which they are most relevant for yield formation.

Soybean yield is defined as the harvested seed dry matter per unit land area. But before delving into the components of yield, it is informative to understand how yield is related to developmental growth phase (Figure 2A). Soybean development is defined by two overlapping phases, a vegetative growth phase and a reproductive phase. The reproductive phase can be divided further into a seed formation period and a seed filling period. Each phase plays a role in yield formation through various physiological processes that determine final yield (Pederson and Licht, 2014). Yield production begins with a vegetative growth phase, starting from emergence (VE) to beginning bloom (R1) (Egli, 2010). In this phase, organs are formed that provide the necessary machinery for producing biomass through photosynthesis and nutrient uptake and assimilation. Node number, a significant yield component, is determined in the vegetative growth phase. Seed number, the most important yield component in soybean, is determined during the next period, which encompasses flowering initiation (R1) to shortly after the beginning of seed filling (between R5 and R6) (Board and Tan, 1995). The third phase is the seed filling period where seed weight is largely determined, starting from the initial lag period of slow seed filling (R5-R6) to the rapid seed filling period (R6-R7) (Egli and Crafts-Brandner, 1996). These overlapping phases provide soybean with significant phenotypic plasticity regarding final yield.

This developmental overview places yield formation into a broader context. The total biomass accumulated by a soybean crop is determined by the amount of solar radiation intercepted during the growing season, when temperatures are suitable for growth, and where the reproductive phase has the greatest impact on final yield. The proportion of total biomass that is converted to seed biomass is the harvest index, which has been increased in soybean and other crops by plant breeders over many years (Evans, 1996). Crop yield improvement and the general improvement in harvest index is the result of changes in underlying physiological processes (Tollenaar and Lee, 2006) that impact the crop during one or more of these developmental phases. Hence, the identification of key physiological processes associated with yield offers a starting point for understanding yield improvement, starting with yield components (Figure 2A) and yield-related components (Figure 2B).

Another important developmental aspect of soybean is that soybean cultivars exhibit one of two predominant growth habits, defined as determinate and indeterminate types (there are also semi-determinate cultivars). The main differences between the growth habits are associated with the main stem, which terminates growth at flowering for determinate types, but not for indeterminate types (Ting, 1946; Tian et al., 2010). Both types have a similar distribution of vegetative and reproductive dry matter accumulation from flowering to maturity and both accumulate about 50% of final vegetative mass between R1 and R5 (although more is accumulated in branches for the determinate types) (Egli and Leggett, 1973). The total time of flower production is shorter for determinate types, but for both types most flowers (∼80%) are produced in a similar time frame (Robinson and Wilcox, 1998). Thus, the defined yield components and yield-related components discussed below can be considered the same for both determinate and indeterminate soybean cultivars.

Finally, the concepts synthesized in this review are relevant for both transgenic and non-transgenic soybean. The transgenes deployed to date confer tolerance to herbicides or resistance to insect pests (ISAAA, 2021). Both types of transgene classes improve plant stress tolerance – herbicide tolerance contributes to decreased competition from weeds in managed environments, while insect resistance reduces damage due to insect pests. For both herbicide tolerant and insect resistant transgenic soybean, the basic aspects of yield physiology are the same as non-transgenic soybean.

## Yield Components

Yield components are the highest-level traits that are directly related to yield and were used as early as the 1920s for analyzing the response of wheat yield to planting density (Engledow and Wadham, 1923). Seed number and weight per seed are the two most fundamental yield components. Ultimately, seed dry matter per unit land area (yield) is determined by the following:


Yield  =  Seed  number  per  unit  land  area  ×  Weight  per  seed

**FIGURE 3:**
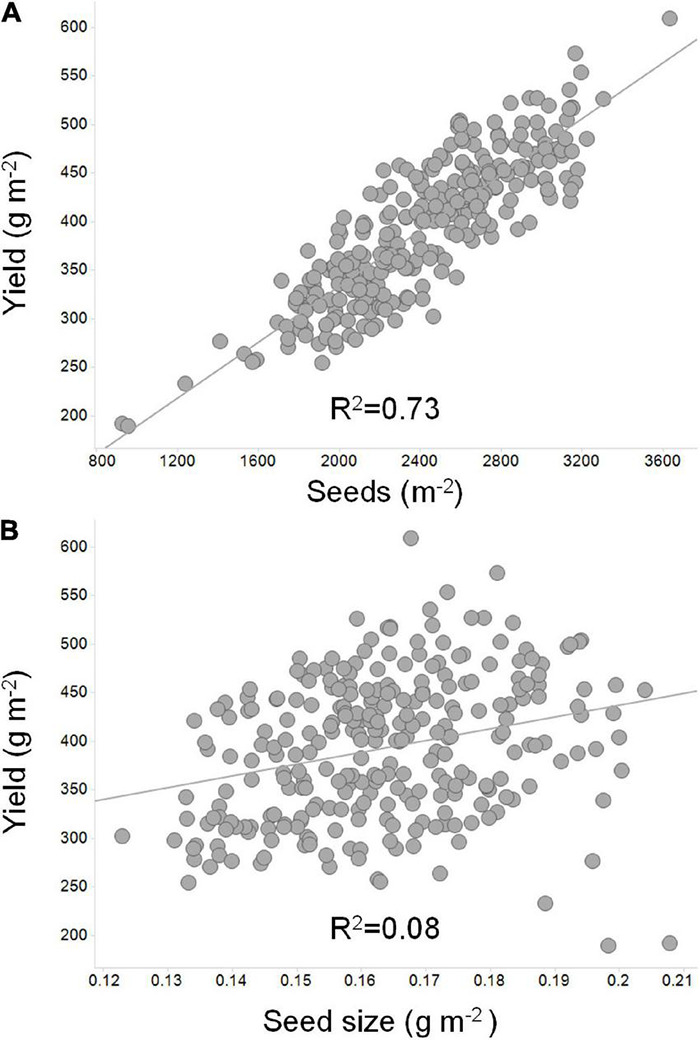
An illustration of the relationship of seed number and seed size to soybean yield. Linear correlations were derived between yield, seed number, and seed size to depict the more significant relationship to yield for **(A)** seed number as compared to **(B)** seed mass. In this example, 40 soybean lines adapted to the Midwest US were grown in replicated field plots at seven locations across Illinois, Indiana, and Missouri (Uniform Soybean Tests: Northern Region 2019; Uniform Test III – Treated Material; https://purr.purdue.edu/publications/3416/1). Seed number per unit area was calculated based on average yield per location divided by average seed size (Nowling and Guohong, 2019).

Of the two components, there is overwhelming evidence that seed number per unit land area is the single most relevant yield component (Board et al., 1999; De Bruin and Pedersen, 2009; Jin et al., 2010; Wei and Molin, 2020). Figure 3 depicts the importance of seed number as compared to seed weight. Seed size, however, can have a significant impact on yield, especially in situations where the seed filling period is long. Cultivars that maximize the number of seeds per unit land area and subsequently fill those seeds to the maximum size will attain their yield potential. Soybean cultivars vary greatly in seed size and seed number per unit land area and compensation between the two yield components is often observed. Essentially, large seeded cultivars have less seeds per unit land area whereas small seeded cultivars have more seeds per unit land area, yet the yield can remain constant in both situations (Hartwig and Edwards, 1970). The importance of this compensation in soybean can be overlooked if focus is placed on demonstrating the impact of a single gene or genetic locus on a single yield component alone.

The typical relationship between seed number per unit land area, weight per seed, and yield is dependent on the growth phases depicted in Figure 2 and is supported by many examples in the literature (Board et al., 1999; Carciochi et al., 2019). As stated earlier, soybean development is defined by two basic overlapping phases, a vegetative growth phase and a reproductive phase. The timing and duration of these phases is highly important in determining final yield. Considering these phases and the general reproductive characteristics of soybean, soybean yield components can be further defined as follows:

1) **Seed number per unit land area = Pod number per unit land area × Seed number per pod**
2) **Pod number per unit land area = Node number per unit land area × Pod number per node**
3) **Seed number per unit land area = Node number per unit land area × Pod number per node × Seed number per pod**
4) **Soybean yield = Node number per unit land area × Pod number per node × Seed number per pod × Weight per seed**

**FIGURE 4:**
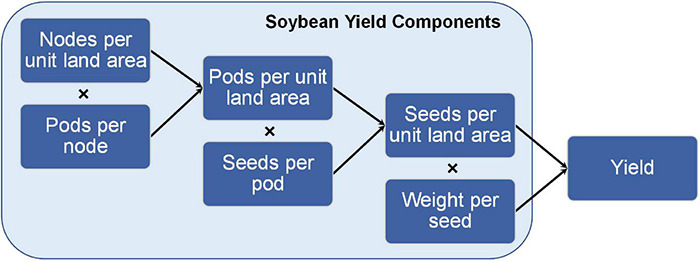
Relationship between soybean yield components and yield. The graphical and mathematical relationship between soybean yield components and final yield on a per unit area measurement.

As indicated in the above equations (Figure 4), the yield components that define soybean yield reflect that yield results from a population of plants growing together in a community (denoted by “per unit land area”) and measurements on that community capture planting density. Similar relationships among yield components can be described on a per plant basis, but great caution must be taken in interpreting such results in terms of yield per unit land area. For example, single plant measurements taken from individual plants grown outside of a community do not meet the above definition of yield. However, carefully conducted field experiments can provide insightful data for both per plant and per unit land area measurements.

## Yield-Related Components vs. Traits

**FIGURE 5:**
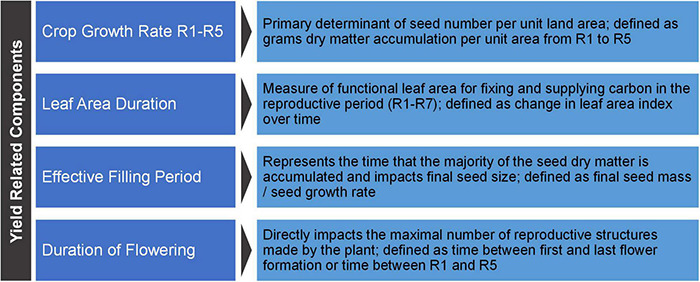
Soybean yield-related components. The basic definitions for soybean yield-related components.

Yield-related components represent the next level below yield components. Yield-related components require a body of definitive data indicating they directly impact yield via impacting one or more yield component. Based on the body of literature, we define four yield-related components for soybean: crop growth rate R1-R5, duration of flowering, leaf area duration (LAD), and effective filling period (Figure 5).

From these few yield-related components spring the myriad of other traits for which there is far less evidence of a direct link to yield. All other traits represent the next level below yield-related components. Here, traits are defined as higher level biological processes that *may* be important, singly or in multiples, in impacting yield and yield-related components. Definitive evidence of a direct yield impact is not a prerequisite for these traits. By contrast, all yield-related components have a body of work directly linking them to yield.

## Crop Growth Rate R1-R5

Crop growth rate R1-R5 is the primary determinant of seed number per unit land area and as such it is the most important yield-related component.

**Crop growth rate R1-R5 is defined as: grams dry matter accumulation per unit land area per unit time (from developmental stages R1 to R5)**

Where R1 is defined as one flower at any node and R5 as when the beans begin to develop (can be felt when the pod is squeezed) at one of the four uppermost nodes with a completely unrolled leaf. This yield-related component can also be expressed on a thermal time basis (grams dry matter accumulation per unit land area per growing degree day).

The partitioning of plant biomass that occurs during R1-R5 and the relationship between crop growth rate R1-R5 and seed number per unit land area in soybean has been established by multiple researchers (Herbert and Litchfield, 1984; Egli, 1993, 2019). Crop growth rate is a function of canopy photosynthesis (or assimilate supply) and it has been demonstrated that increasing canopy photosynthesis during R1-R5 by high CO_2_ treatments or reflector treatments significantly increases the number of seeds per unit land area (Hardman and Brun, 1971; Schou et al., 1978; Mathew et al., 2000).

Canopy photosynthesis is the net amount of carbon fixed by a plant population per unit land area per unit time. Net canopy photosynthesis is determined by the balance between canopy photosynthesis and dark respiration, the two highest level traits for crop growth rate R1-R5. Dark respiration has historically been considered as the sum of “maintenance” and “growth” respiration (Loomis and Amthor, 1999). However, these two components are theoretical and not measurable. The rate of dark respiration for crop canopies can be up to half the rate of canopy photosynthesis and high nighttime temperatures are often associated with decreased yield (Egli and Wardlaw, 1980; Wilson and Jones, 1982; Seddigh and Jolliff, 1984; Albrizio and Steduto, 2003). Even considering just these two traits, one can begin to derive multiple additional traits that can influence both canopy photosynthesis and dark respiration (e.g., canopy architecture, pod distribution, water uptake, stress tolerance, lodging, etc.).

## Duration of Flowering

There are two possible definitions for duration of flowering. They are (1) time between growth stages R1 and R5 and (2) time between the first and last flower observation. This yield-related component does not have as much evidence of a definitive relationship with yield as for the other yield-related components. The most compelling evidence is contained in a series of papers where imposition of long day treatments around R3 significantly increased node, pod, and seed number per unit land area (Kantolic and Slafer, 2001, 2005, 2007). When supplemental light was maintained from R3 through maturity, the length of the seed filling stage was reduced and delayed; therefore, seed filling commenced in less favorable environmental conditions, resulting in reduced seed weight (seed size was reduced ∼20%, but seed number was increased >75%). While not directly changing the length of the flowering period, the studies indicate that the timing of flowering with favorable growing conditions can impact yield (Kantolic et al., 2007; Nico et al., 2015). A few other publications directly associate flowering to seed number or yield. Dybing found a positive relationship between flowering period (time between first and last flower) and seed yield per unit land area (Dybing, 1994). Additionally, a relationship between the length of flowering and pod set and seeds m^–2^ was found when comparing yield from various soybean cultivars planted at an early and late planting date (Egli and Bruening, 2000).

The duration of flowering is mainly influenced by photoperiod and temperature. In a long-term soybean yield study, Cooper (2003) found a positive correlation between yield and warm spring temperatures that induced early flowering (Cooper, 2003). Cooper suggested that an extended reproductive period was responsible for the increased yield. This is corroborated by modeling work from Kantolic et al. (2007), that suggests increased yield could be realized through early flowering and an extended post-flowering phase without changing the total cycle length, such that the seed filling period would not be shifted to suboptimal growing conditions later in the season. Their modeling suggested that earlier flowering should increase yield across a broad range of latitudes and environmental conditions. This is supported by early planting studies, which show early planting shortened the vegetative stage and lengthened the reproductive stage (Rowntree et al., 2014).

These studies demonstrate that duration of flowering is critical, as is the alignment of that period with growing conditions. The impact of reproductive timing on yield is evident when there is a mismatch between the reproductive phase and the environment. Soybean is a short-day plant and is sensitive to the photoperiod in which it is grown. The yield of soybean is dependent on adaption to target latitude (Zhang et al., 2007). An example of the critical nature of adaption and timing is the long juvenile (LJ) trait. Soybean grown in low-latitude regions historically suffered from early flowering, early maturity, and low yield. The LJ trait delays flowering, allowing sufficient vegetative growth prior to flowering under short-day conditions (Destro et al., 2001). A major locus for this trait, *J*, was cloned and shown to encode an *EARLY FLOWERING 3* (*ELF3*) ortholog involved in the control of flowering (Lu et al., 2017). Introduction of the LJ trait has allowed successful expansion of soybean production into low-latitude regions and provides another example of the importance of developmental timing with the environment.

Taken together, these studies point toward the importance of the duration of flowering. For this yield-related component, both the length of the flowering period and the timing of this period with the environment are critical in maximizing yield. This physiological knowledge helps explain why early planting can increase yield, as it lengthens the reproductive duration (Rowntree et al., 2014). Conversely, late planting fails to properly align optimal growth conditions with each developmental stage, resulting in decreased yield (Kessler et al., 2020).

## Leaf Area Duration

LAD is equated with the amount and photosynthetic ability of canopy leaf area across the time of grain filling. LAD is important to some extent for pod and seed number per unit land area, but it is a major determinate of final seed size.


$$\begin{array}{l} \text{Leaf Area Duration is defined as:}\\ (\frac{\text{Leaf Area Index T2 + Leaf Area Index T1}}{2})\;\times \;(\text{T2-T1}) \end{array}$$

Here, leaf area index (LAI) is defined as the m^2^ leaf area m^–2^ ground area and T1 and T2 are time of sampling and units are days (Watson, 1947; Hunt, 1990). The assumption made is that the leaf area is “functional” and thus maintaining some rate of canopy photosynthesis. LAD can be calculated for any or all developmental stages, but in the context of yield, the relevant measurement would be at intervals from R1-R7 (beginning flower to physiological maturity where 50% of pods are yellow). Therefore, functional LAD is best measured by determining canopy photosynthesis from R1 to R7.

There exist different methods to determine the relationship between LAD and yield. For example, Liu et al. (2005) measured LAD based on leaf area index over time, demonstrating that high yielding cultivars were superior to medium and low yielding cultivars throughout the grain filling period. Another way to measure this relationship is via measurement of canopy photosynthesis during the grain filling period (Wells et al., 1982). In this experiment, canopy photosynthesis was highly correlated with seed yield per unit land area. Other experiments measuring leaf area index corroborate that newer soybean cultivars (which have higher yield) maintained a greater LAI for a longer duration than old cultivars (Kumudini et al., 2001) and this impact of LAD on yield holds true across legume crops (Laing et al., 1983).

Traits that impact LAD would be expected to have an impact on the functional canopy during grain filling. Extending this period (measured on the canopy) should increase assimilate availability for seed filling and hence increase yield. Traits that affect LAD should impact the canopy, assimilate supply, and ultimately seed weight. This can include traits such as leaf traits (e.g., morphology, orientation), carbon fixation, canopy architecture, stay green/senescence, carbon partitioning, and abiotic/biotic stress tolerance.

## Effective Filling Period (Seed Filling Period)

Effective filling period is the period of time during which a seed is accumulating dry matter at a linear rate and it is associated with maximum seed size.


$$\text{Effective Filling Period(EFP)is defined as: }\frac{\text{Final seed size(mg)}}{\text{Seed growth rate}}$$

Here, seed growth rate is the seed dry matter (mg) gain per day and is measured during the time of linear seed dry matter accumulation. The final seed size is measured at R8 (full maturity). Effective filling period can also be based on growing degree days. By definition, a longer effective filling period will be associated with attainment of maximum seed size. The effective filling period is different from the total reproductive period measured from flowering to R8 or the total seed filling period measured from R5 to R8 (Daynard et al., 1971; Salado-Navarro et al., 1985; Egli, 2017). This yield-related component is closely related to LAD, but the measurement is on the seed. There is an inverse relationship between seed growth rate and seed number.

The traits most associated with effective filling period are largely associated with the canopy during grain filling, particularly during maximum seed dry matter accumulation. Light interception is one of the main traits and evidence suggests soybean needs to achieve 95% light interception for optimal yield (Shibles and Weber, 1966; Egli, 1988; Board et al., 1992; Kumudini et al., 2001). Examples of the importance of light interception include studies with shade treatment that reveal that flower and pod number are impacted by shading stress (Jiang and Egli, 1993). Unlike corn (where maximal kernel number is fixed early in reproduction), soybean has strong reproductive plasticity, in part due to the overlap of pod formation, seed set, and seed filling periods. This overlap allows shading or thinning to have a strong impact on seed number. When faced with reduced light interception during the early reproductive period, soybean reduces seed number so that when seed filling period begins, seed size is unaffected. Traits such as canopy architecture, leaf angle, plant height, stay green, temperature, and others can all impact the ability of the canopy to capture light.

## Summarizing Yield-Related Components

The above yield-related components (except for duration of flowering) can be related to yield components mathematically and conceptually as follows:


Yield  =  Seed  number  per  unit  land  area  ×  Weight  per  seed

Where:


$$\text{Seed number per unit land area }=\;\frac{\text{Assimilate supply}}{\text{Seed growth rate}}$$

And:


Weight  per  seed  =  Seed  growth  rate  ×  Effective  filling  period

**FIGURE 6:**
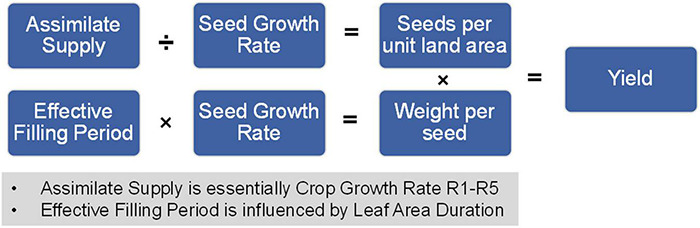
Relationship between soybean yield-related components and yield. The mathematical relationship between soybean yield-related components and final yield on a per unit area measurement.

In these equations, assimilate supply is analogous to crop growth rate R1-R5 or canopy photosynthesis and effective filling period is influenced by leaf area duration (LAD also impacts assimilate supply). This set of equations is summarized in Figure 6 and reveal the importance of the yield-related components. Seed number per unit land area and possibly seed weight can be impacted if the reproductive growth phase is not optimally aligned with growing conditions. Thus, the yield-related components are the embodiment of the yield components.

## The Importance of the Reproductive Period

Returning to soybean developmental stages, we find all yield-related components are directly associated with the reproductive phase of crop growth. Hence, this period of growth is the most important phase for achieving full yield potential. Barring significant compensation, any physiological process that positively impacts the yield-related components is expected to impact yield. Vegetative phase traits such as canopy or root architecture, flooding tolerance, cold tolerance, drought tolerance, and seed quality can impact final yield, but ultimately the impact from these traits will be realized via one or more of the yield-related components. As such, it is important to understand both yield-related components and their underlying traits (Xavier et al., 2017a).

As stated earlier, soybean intrinsic yield potential is high and growth limiting factors (largely stress) are the major limitations to high yield across the broader regions of soybean production. Stress can negatively impact growth and development at all growth stages, but stress during reproductive growth is generally more detrimental to yield and often leads to significant yield loss (Board and Kahlon, 2011; Siebers et al., 2015; Monzon et al., 2021). For example, soybean is highly sensitive to drought during reproduction. If drought occurs during flowering, soybean responds by producing fewer flowers, pods, and seeds (Sionit and Kramer, 1977), with the main impact on pod number rather than seeds per pod (Board and Kahlon, 2011). Soybean can recover from brief episodes of stress during flowering because maximal yield potential is not based on the successful development of all fruiting bodies. However, severe stress or long periods of stress will have a greater impact on yield, as does stress during the seed filling period (Anda et al., 2020; Veas et al., 2021). Given the great propensity of stress to decrease yield, mitigating the impact of stress during the reproductive period is a key target for crop improvement. The impact of stress elimination is clear, as irrigation or fungicide, herbicide, insecticide, and fertilizer application are well known methods of increasing yield through stress elimination in high input agronomic systems (Cafaro La Menza et al., 2017; Greer et al., 2020).

## Leveraging Yield Physiology in an Era of Precision Breeding

With this overview of soybean yield components, a key question that arises is, how does one leverage knowledge of soybean yield-related components for crop yield improvement? While yield improvement has occurred over time due to improved genetics, agronomics, chemistry, and biotechnology traits, each improvement ultimately impacted one or more yield-related component. And while new breeding technologies continue to push yield forward, this is largely, if not entirely, done without purposely selecting for a specific yield-related component or underlying trait. For example, genomic selection can accelerate genetic gain for yield (Stewart-Brown et al., 2019). But the use of genomic selection on yield-related components is currently impractical, due to the complexity and effort required to measure such traits at scale. Another method for improvement is use of molecular precision breeding technologies that can efficiently introduce genetic variation for a specific desirable trait (Chen et al., 2019; Gao, 2021). Here, our focus is on the use of precision techniques for improvement of yield-related components and component traits.

To successfully alter soybean traits in a precise manner, it is useful to first define precision breeding technologies and their requirements. Precision breeding encompasses several methods that benefit crop breeding and has two basic criteria – the technology focuses on a specific trait and requires genetic targets to improve that trait. The evolution and application of precision breeding technologies is best considered in the context of conventional breeding (Anderson et al., 2019; Ahmar et al., 2020). Conventional plant breeding methods are efficient at improving yield through the introgression of desired alleles in crossing programs, but this typically requires lengthy timelines to achieve superior performance with all desired traits. Precision breeding methods help shorten breeding timelines by utilizing specific genetic elements (genes and loci) that confer improved trait performance. For example, by taking advantage of genetic linkages, DNA markers for desirable traits can be used to efficiently introgress these traits via marker assisted selection (MAS) (Collard and Mackill, 2008).

Transgenic technology represents another form of precision breeding, where expression cassettes for genes of interest are inserted into the genome to confer a desirable trait, such as insect resistance or herbicide tolerance (Kumar et al., 2020). Specifically, transgenesis is defined as the transfer of genes between non-crossable species, while cisgenesis refers to specific gene transfer from a sexually compatible donor plant (Schouten et al., 2006) and intragenesis to the transfer of genes from a crossable species which contain novel combinations of naturally occurring genetic elements (Rommens, 2004). With transgenics, desired traits can therefore be directly introduced from any species, without the transfer of unwanted elements linked to the desired trait (i.e., linkage drag).

More recently another precision breeding technology, genome editing, has garnered much attention (Songstad et al., 2017; Knott and Doudna, 2018). Genome editing relies on the delivery of genome editing components into plant cells to specifically modify native targets in the genome, creating targeted insertions and/or deletions. The ability to create specific, targeted genetic changes has resulted in the high level of interest in this technology. It is still too early to know whether genome editing will ultimately provide large gains in breeding speed, efficiency, or yield gains (Lyzenga et al., 2021). But, if genome editing is to successfully impact yield, any edit must impact a yield-related component. Hence, it is useful to map out the requirements of editing (or any precision breeding technology) for crop improvement.

Precision breeding via editing has several requirements. These include knowledge of (1) what trait target one desires to impact, (2) what gene targets should be edited to achieve a change and what edits to make, (3) which combination of edits are required to obtain the desired outcome, and (4) how to monitor for the desired trait impact in the field. To highlight the use of physiological knowledge for crop improvement via precision breeding, we will focus on each step in turn.

## Identifying and Selecting Traits to Impact Yield-Related Components

Precision breeding-based improvement requires the selection of traits which, when modified, should impact yield-related components. As described earlier, each yield-related component can be further related to numerous traits that contribute to the final expression of yield through the yield-related components. The identification of target traits can be derived from prior knowledge, hypothesis, or experimentation. And while we focus on the higher level yield-related components, others have delved deep into the various traits that underlie yield-related components, including stress tolerance traits (Ramachandra et al., 2015).

Experimentation can address yield component relationships and their contribution to yield through yield component analysis (Xavier et al., 2017a). However, this has proved challenging, as such studies have provided mixed results. For example, in one study the total number of pods was determined to have a significant linear correlation with grain yield (Ferrari et al., 2018), which is logical as pod number and node number are known yield components. However, another study using the SoyNAM population determined that pod number and node number had low heritability and did not have a strong genetic correlation to yield (Xavier and Rainey, 2020). While variation in methods, data collection, and analysis technique contribute to differences, more fundamental reasons exist for why yield trait component analysis has historically been limited. This is addressed by Egli, where he describes several reasons for why historic efforts focusing on yield components did not always improve our understanding of yield or aid in breeding efforts (2017). First, compensation between traits complicates any experiment. Second, simple statistical correlations without consideration of underlying physiological processes are not always useful. And third, components measured on a per plant and not a per unit area basis may create confusion as components can be sensitive to plant population. For example, pods per plant varies inversely with plants per unit area, so pods per unit area remains constant (Egli, 1988).

Despite challenges, carefully designed and conducted field experiments coupled with physiological knowledge can provide valuable insight into trait impact on yield (Sinclair and Purcell, 2005). The study of how simpler traits relate to yield components or yield-related components allows one to identify both important traits and the genes that contribute to trait expression. Additionally, as more sophisticated analysis techniques (such as machine learning and related methods) and computer-based phenotyping become available, the possibility for unraveling such complex relationships increases (Ramos-Giraldo et al., 2020; Riera et al., 2021; Yoosefzadeh-Najafabadi et al., 2021).

In the end, if a yield relevant trait is selected (one applicable to plant growth in a community and which limits yield potential), then improvement is possible. This is essentially what occurred via introduction of the Green Revolution genes in wheat and rice (Hedden, 2003). Targeted reduction in plant height reduced lodging and improved response to nitrogen application. Depending on the timing and severity, lodging can potentially impact all yield-related components (Egli, 2017).

Plant height is also relevant for soybean, as newer higher yielding cultivars tend to be shorter and have reduced lodging (Specht and Williams, 1984; Rincker et al., 2014). Yet given the growth characteristics of soybean, where pods form at multiple nodes, simply reducing plant height alone is unlikely to directly increase yield. Hence, this is just one example of how the knowledge of traits and their relationship to yield components is valuable in trait selection and modification. It also highlights why it is important to measure the trait, yield-related components, and yield components to understand how a trait relates to yield.

## Gene Target Identification for Yield-Related Component Improvement

Once a trait is chosen for its probable impact on a yield-related component or yield component, loci controlling that trait need to be identified and brought into a breeding population. Or, in the case of editing, target genes are required. As such, known links between targets (genes) and phenotypes (traits) in crop plants grown under agronomic conditions are essential (Sinclair and Purcell, 2005). For many simply inherited traits, several potential targets may already exist in the literature. One example of this comes from work by Nguyen et al. (2020) where a soybean ortholog of an Arabidopsis gene (*KIX8*) was identified and found to underlie a major seed weight QTL. Genome editing was then used to increase soybean seed size by knocking this gene out (however, seed number was reduced). Additional examples include results from rice, where individual and combinatorial knock outs of three yield-related QTL genes identified to negatively regulate grain size (*OsGS3)*, grain width and weight (*OsGW2)*, and grain number (*OsGn1a*), revealed varied changes to seed number and seed weight on a per plant basis (Zhou et al., 2019). Traits underlying yield-related components have also been studied with editing. A series of higher-order mutants in soybean of the *GmSPL9* gene family exhibited varying changes in nodes per main stem, total node number, branch number, and 50-day-old plant dry weight (Bao et al., 2019). And in maize, the *ARGOS8* gene, a negative regulator of ethylene response, was shown to impact maize yield under drought stress when the native promoter was replaced with the maize *GOS2* promoter (Shi et al., 2017). While these examples depended on prior knowledge, many traits either have not been studied or have largely been studied in controlled conditions on individual plants, not in field relevant communities.

As editing is dependent on changes to native genes, knowledge of gene-to-trait interactions relevant to crop growth under agronomic conditions is critical. Since multiple traits and loci interact and many loci can underlie a trait, an understanding of genetic architecture provides a tremendous advantage in applying precision approaches. Hence, crop-based knowledge of gene-to-trait associations (from QTL mapping, GWAS, candidate gene characterization, breeding data, or other mapping methods), especially those conducted under different agronomically relevant environments is (and will likely remain) a rate limiting factor for fully realizing the potential of precision breeding approaches (Scheben and Edwards, 2018; Tibbs Cortes et al., 2021). Additionally, in order to select the genes from candidate loci most likely to positively impact a trait, it is likely that the integration of varied datasets from multiple disciplines (breeding, physiology, genetics, molecular studies, etc.) will be needed to successfully select key precision breeding targets (Zivy et al., 2015; Zhou et al., 2020). As such, one goal of the larger plant biology community should be to support the integration of plant breeding, physiology, genetics, and molecular knowledge to fully realize precision breeding techniques (Liu et al., 2021; Varshney et al., 2021).

Linking a gene to a trait is only the first step. If editing is to succeed, one needs to know both the target gene and the desired change to create. Yet the challenge is, in most cases, the exact change to create is not known. One possibility to overcome this challenge comes from studies in tomato, where careful selection and manipulation of traits via genome editing altered fruit size, inflorescence branching, and plant architecture (Rodríguez-Leal et al., 2017). Rather than spending upfront effort identifying the exact change required, the authors combined the best of genome editing with forward genetics. Using genome editing tools to create random novel allelic variation at the gene of interest, they then screened newly edited plants for the desired trait. Only then did the researchers determine which genetic change was created and necessary. In this case, changes in gene regulatory sequences were generated to create the allelic variation. The authors were successful in altering all three traits, demonstrating the feasibility of impacting traits via editing regulatory elements. This establishes that such a screening strategy is an efficient way to determine which genetic changes lead to favorable phenotypes. Importantly, examples exist for regulatory sequence changes impacting important soybean domestication traits, such as seed shattering (Dong et al., 2014), semi-determinate growth (Ping et al., 2014), and pod color (He et al., 2015), indicating that this strategy is feasible for soybean.

An alternative method for specific edit identification is hypothesis generation and experimentation to generate detailed molecular, genetic, and/or biochemical knowledge on gene function. For example, the body of work surrounding the plant lipid biosynthetic pathway (He et al., 2020) provides multiple potential targets, some of which (*FAD2-1A*, *FAD2-1B*, *FAD3A*) have been successfully edited in soybean (Demorest et al., 2016). Yet had these targets not been known, directed experimentation would have eventually led to these targets. Given the breadth of molecular and biochemical techniques, reverse genetics approaches can be quite fruitful, but require time. The use of varied techniques to understand gene function and expression supports the premise that multiple disciplines will be required to fully implement precision breeding techniques such as genome editing (Pazhamala et al., 2021). Both forward (targeted random mutation) and reverse (gene function experimentation) methods are likely to identify impactful edits, hence the pros and cons of each approach must be considered before a researcher decides which path to follow.

## Measuring the Impact of Precision Breeding on Traits and Yield-Related Components

After a trait is selected, a target identified, and specific edits created, the impact of the changes need to be quantified. This is likely to first occur at the individual plant level. Yet knowledge of yield-related components dictates that single plant performance is informative but must translate to community performance. While it is feasible to screen a handful of edited lines in field trials at one location for improvement in yield-related components, such an endeavor becomes daunting at scale. That said, it would be extremely valuable to screen populations of edited plants or even breeding populations for changes in specific traits or yield-related components at scale, in a field, at the canopy level. So, how does one measure specific traits or even yield-related components in field populations at scale?

One answer lies with modern digital tools and high-throughput phenotyping technologies. With these, quantification of many morphological and physiological traits could feasibly be collected in breeding programs. One high-throughput field phenotyping platform is unmanned aerial systems (UAS). UAS can rapidly assess thousands of plots in a field with high spatial and temporal resolution. For example, average canopy coverage as measured by UAS can be used for selection, alone or in combination with yield, in early stages of a soybean breeding pipeline (Moreira et al., 2019). Canopy measurements can also be used as approximations of yield-related components. Average soybean canopy coverage as measured by UAS was used to estimate crop growth rate and found to be highly heritable, having a high genetic correlation with yield (Xavier et al., 2017b). And early season canopy coverage has also been used to improve the predictive accuracy of yield in genomic prediction models (Jarquin et al., 2018). Finally, a high-throughput phenotyping platform using 3D reconstruction technology was successfully used to quantify physiological growth dynamics and biomass estimations of soybean to identify soybean varieties that possess maximum growth rate (Zhu et al., 2020).

Despite such advances in above canopy measurements, the complexity of soybean growth in populations remains an obstacle in the evaluation of yield components such as node number and pod number, which are still collected via traditional manual counts. Such measurements are slow, labor intensive, and destructive. Despite this, researchers are rising to the challenge. In recent years, great progress has been made in developing automated data collection platforms such as ground robots with digital sensors creating frameworks to estimate seed yield at the canopy level from breeding plots (Gao et al., 2018; Parmley et al., 2019). Additionally, machine learning approaches have been used to estimate in-season seed yield using a deep learning-based multi-view image fusion framework (Riera et al., 2021). Here, a core model for pod detection and localization was developed and subsequently deployed. Yield estimation was conducted using a robotic platform for pod counting of individual plots in real-time. Such technologies deployed in a breeding pipeline could significantly improve the capability to obtain high quality yield component data, overcoming the obstacle of manual data collection. Such efficient measurements of yield components, traits, stress response, or even eventually yield-related components will be required to scale-up phenotyping for large-scale editing efforts (Ramos-Giraldo et al., 2020).

## Discussion

Precision breeding has the potential to be a valuable approach to improving yield and yield-related components and gene editing has promise in this regard that can be realized through leveraging multiple disciplines. In reviewing the steps required for improving yield-related components via genome editing, this need for multiple disciplines becomes clear, revealing the necessity for new innovations spanning the entire plant biology community. Still, much work is required to ensure the success of genome editing enabled yield-related component improvement.

Both new technologies (machine learning, phenotyping platforms) and biological datasets (trait-to-gene linkages and molecular knowledge) need to be developed. Genome editing also faces technical challenges for implementation of the technology itself, including the multiplexing of edits, editing efficiency, and editing component delivery into cells, which are discussed in detail by others (Chen et al., 2019; Mao et al., 2019; Yang, 2020; Gao, 2021). Finally, there are practical considerations for how editing is incorporated into existing breeding pipelines (Jung et al., 2018; Hickey et al., 2019).

These technical hurdles are worthy challenges to address but complicated further by the existence of compensation. Compensation is the great propensity of the plant to offset, such that an increase in one yield component is accompanied by a decrease in another (Evans, 1996; Egli, 2017). And unlike crops such as maize, soybean has a long flowering period, allowing the plant even greater time to compensate (Dybing, 1994; Egli, 2010). These compensatory relationships likely stem from evolutionary factors, as plants evolved to make the most of available resources to reproduce and ensure survival of the next generation. As opposed to evolution, the goal of a breeder is to develop plant varieties that produce the highest yield when grown in monoculture under strictly managed agronomic conditions (Weiner, 2019).

Another biological challenge for precision breeding is the quantitative nature of yield and yield components. The multigenic nature of yield-related components means that the contribution of many loci across the genome and their interactions over the course of a growing season determine final yield. For any genome edited plant, the interaction with these loci both across germplasm and environments must be evaluated to determine the overall agronomic impact on both yield and yield-related components.

Technical and biological challenges notwithstanding, public perception and the global regulatory climate must also be considered. Global guidelines for genome editing product release vary by country and remain an area of active discussion (Friedrichs et al., 2019; Menz et al., 2020). And while genome editing can create the same types of genetic variation naturally found in any species, this distinction does not guarantee favorable public acceptance (Ishii and Araki, 2016). As such, there is a great need for the plant biology community to strongly advocate for genome editing technology.

Despite the challenges, the plant biology community should leverage new technologies to achieve the goal of improving yield-related components and underlying traits. The promise of continued yield improvement for new soybean varieties is important to sustainably produce more with less – less input with fewer acres in the face of climate change. To reach this goal, the use of all available breeding tools will be required. The potential of creating novel genetic variation beyond that present in natural populations by precise modification of key yield traits represents an exciting new era for crop improvement.

If precision breeding and the molecular work on the understanding of gene function is to be translated into yield gains, it must be kept in mind that yield components, yield-related components, traits, and G × E × M interactions ultimately lead to yield. To further this endeavor, this top-down review of yield physiology provides a high-level overview of yield physiology, including context for how yield-related components relate to both yield components and crop development. This knowledge is the foundation on which to build trait selection, target discovery, and crop phenotyping efforts. And while our focus here is soybean, the basic concepts hold true for other crops and can similarly inform endeavors in those crops as well. In keeping the basic yield physiology concepts summarized here at the forefront, the plant biology community increases the odds of successfully manipulating specific individual physiological traits (for which there is far less field-based evidence for a direct link to yield) and achieving the goal of using precision breeding technologies for yield improvement.

## Author Contributions

JV, WL, PO, SC-B, JP, and NC contributed to the background literature review, writing, and editing of this manuscript. All authors contributed to the article and approved the submitted version.

## Conflict of Interest

The authors of this manuscript are present or former employees of BASF Corporation or a BASF subsidiary company.

## Publisher’s Note

All claims expressed in this article are solely those of the authors and do not necessarily represent those of their affiliated organizations, or those of the publisher, the editors and the reviewers. Any product that may be evaluated in this article, or claim that may be made by its manufacturer, is not guaranteed or endorsed by the publisher.
